# A cross-sectional study of the association between circulating sex hormone-binding globulin levels and selected adipokines in women with polycystic ovary syndrome

**DOI:** 10.3389/fmed.2023.1183961

**Published:** 2023-07-25

**Authors:** Dariusz Ciura, Aleksander Jerzy Owczarek, Grzegorz Franik, Piotr Kocełak, Leszek Markuszewski, Paweł Madej, Jerzy Chudek, Magdalena Olszanecka-Glinianowicz

**Affiliations:** ^1^Health Promotion and Obesity Management Unit, Department of Pathophysiology, Faculty of Medical Sciences in Katowice, Medical University of Silesia, Katowice, Poland; ^2^Department of Gynecological Endocrinology, Faculty of Medical Sciences in Katowice, Medical University of Silesia, Katowice, Poland; ^3^Pathophysiology Unit, Department of Pathophysiology, Faculty of Medical Sciences in Katowice, Medical University of Silesia, Katowice, Poland; ^4^Faculty of Medical Sciences and Health Sciences University of Humanities and Technology, Radom, Poland; ^5^Department of Internal Medicine and Oncological Chemotherapy, Faculty of Medical Sciences in Katowice, Medical University of Silesia, Katowice, Poland

**Keywords:** SHBG, adipokines, nutritional status, polycystic ovary syndrome, adipose tissue

## Abstract

**Introduction:**

Changes in the proportion of pro-inflammatory and anti-inflammatory adipokines may reflect the accumulation of lipids in the liver and the development of insulin resistance. Both liver steatosis and insulin resistance result in decreased sex hormone-binding globulin (SHBG) synthesis. This study aimed to analyze associations between circulating SHBG and adipokines levels in women with polycystic ovary syndrome (PCOS).

**Material and methods:**

A cross-sectional cohort study involved 87 women with phenotype A of PCOS (39 normal weight and 48 obese). Body mass, height, and waist circumference were measured, and BMI was calculated. In addition, body composition was assessed using the bioimpedance method. Serum SHBG levels and plasma apelin-36 and apelin-12, adiponectin, leptin, omentin-1, and RBP-4 were determined by using the ELISA method. The participants were divided into subgroups with SHBG concentrations above and below this lower limit [*N* = 35 (40.2%) and *N* = 52 (59.8%), respectively].

**Results:**

The median adiponectin, apelin-12, and apelin-36 levels were significantly lower, and leptin levels were significantly higher in the subgroup with low SHBG levels than that in the subgroup above the lower limit of the reference range, while there were no differences in median omentin-1 and RBP-4 between the study subgroups. There were positive correlations between SHBG and omentin-1, adiponectin, apelin-36, and apelin-12 levels, as well as negative correlation with leptin levels. However, after adjustment by BMI, waist circumference, and body fat percentage, only the association between SHBG and omentin-1 remained significant.

**Conclusion:**

Our results show associations between circulating SHBG and adipokine levels in women with PCOS and support the role of hormonal dysfunction of the adipose tissue in the pathogenesis of PCOS.

## Introduction

Polycystic ovary syndrome (PCOS) is characterized by numerous endocrine disturbances including low SHBG levels in the circulation. The liver is the main site of SHBG synthesis, stimulated by estrogens and inhibited by insulin and androgens. One of the pathomechanisms of hormonal disturbances specific to PCOS is insulin resistance. Hyperinsulinemia is a mechanism compensating insulin resistance and decreasing SHBG synthesis in the liver (1–3). A decreased SHBG level results in an increase in the concentration of free estrogens and androgens, which in turn affects the activity of the hypothalamic–pituitary–ovary axis (4).

The regulation of SHBG synthesis seems to be more complex. Some studies have suggested that adipokines may also regulate SHBG synthesis. However, data on this issue are limited. An experimental study showed that adiponectin may stimulate SHBG synthesis in human hepatic cell cultures by the activation of the AMPK pathway. However, this process was inhibited by increased lipogenesis and decreased fatty acid oxidation (5). The association between circulating SHBG and adiponectin levels has also been reported in various populations. A positive correlation between SHBG and adiponectin levels regardless of gender, BMI, and fat mass was shown in a study that included 785 adolescents aged 12–16 years (6). Similar results were obtained in a pediatric population of 160 girls and boys in the peri-pubertal age (7), 334 adults aged 50–85 years (8), 1, 881 midlife (9), 634 postmenopausal women (10), and 614 men aged over 40 years (11).

Leptin, another adipokine, has been described to be associated with circulating SHBG levels. An experimental study showed that hyperleptinemia activates caspase-1 and−3 in rat hepatocytes, triggering hepatic pyroptosis resulting in the progression of NAFLD (12). Thus, leptin may inhibit SHBG synthesis in hepatocytes. In line with this hypothesis, some studies showed a negative correlation between SHBG and leptin levels. This association was confirmed in 723 adolescents aged 12–16 years (both in girls and boys) (13), 634 postmenopausal women (10), 614 men over 40 years old (11), and 210 infertile men (14). Currently, there are no published data on the relationship between the concentration of adipokines such as omentin-1, apelin, and retinol-binding protein 4 (RBP-4) and SHBG levels.

As mentioned above, hormonal disturbances observed in PCOS women include decreased circulating SHBG levels that may enhance other hormonal disturbances (1). The hormonal function of visceral adipose tissue and circulating adipokines levels in PCOS women are closely related to the nutritional status (15–18). However, there is a lack of studies assessing the relationships between SHBG and adipokines levels in PCOS women. It has been shown that changes in the proportion of pro-inflammatory and anti-inflammatory adipokines are associated with lipid accumulation in the liver and the development of insulin resistance (19). Therefore, this study aimed to analyze associations between circulating SHBG and adipokines levels in PCOS women.

## Materials and methods

A cross-sectional cohort study involved 87 women with PCOS with a mean age of 26 years (36 with normal weight and 51 with overweight or obesity) with self-reported stable body weight during the last 3-month period, recruited from among the patients of the Department of Endocrinological Gynecology between 2017 and 2019. PCOS was diagnosed based on the Rotterdam ESHRE/ASRM criteria from 2003 (20). Only women who possessed all three of the Rotterdam criteria were enrolled. The exclusion criteria included thyroid dysfunction, Cushing's syndrome, 21-hydroxylase deficiency, androgen-secreting tumor, decreased ovarian reserves, type 1 or 2 diabetes, smoking and alcohol abuse, and any pharmacological therapy. The study was conducted after obtaining the informed consent of each participant, based on the study protocol approved by the Bioethical Committee of the Medical University of Silesia (CBN/0022/KB1/97/21).

Body weight, height, and waist circumference were measured, and body mass index (BMI) was calculated according to the standard formula. In addition, body composition was assessed using the bioimpedance method (Bodystat 1500, Douglas, Isle of Man). Between 3 and 5 days of the women's menstrual periods, 15 ml venous blood samples were withdrawn in the morning between 8.00 and 9.00 a.m. after an overnight fast (14 h) and were collected according to the recommendations of the kit manufacturers. Plasma and serum aliquots were frozen and stored at −70°C.

### Laboratory procedures

Serum SHBG was determined using the ELISA method (DRG Instruments GmbH, Marburg, Germany) with a limit of quantification (LoQ) of 0.2 nmol/l and an intra- and inter-assay of 5.3 and 9.0%, respectively.

The ELISA method was also used for measurements of plasma apelin-36 and apelin-12 (Phoenix Pharmaceuticals, Burlingame, USA), leptin (TECOmedical AG Sissach, Switzerland), adiponectin (TECOmedical AG Sissach, Switzerland), omentin-1 (DRG Instruments GmbH, Marburg, Germany), and RBP-4 levels (Phoenix Pharmaceuticals, Burlingame, USA) with the LoQ of 0.08 ng/ml, 0.11 ng/ml, 0.2 ng/ml, 0.6 ng/ml, 0.5 ng/ml, and 2.17 ng/ml, respectively. Moreover, intra- and inter-assay coefficients of variations were 4.6% and 4.6% for apelin-36; 6% and 7% for leptin; 5% and 6% for adiponectin; 3.7% and 4.6% for omentin-1; and 5.0% and <14.0 for RBP-4.

### Data analysis

Normal weight was defined as having BMI between 18.5 and 24.9 kg/m^2^, overweight 25–29.9 kg/m^2^ and an obesity value of >30.0 kg/m^2^. The participants were divided according to the lower limit of the SHBG concentration laboratory's reference range for women aged 18–50 years (<26.1 nmol/L) into a subgroup with concentrations above and below the limit.

Increased FAI (total testosterone divided by the SHBG level, multiplied by 100) was defined as a value of >5.0.

### Statistical analysis

Statistical analysis was performed using STATISTICA 13.0 PL (TIBCO Software Inc., Palo Alto, CA, USA) and StataSE 13.0 (StataCorp LP, TX, USA). Statistical significance was set at a *p*-value of <0.05. All tests were two-tailed. Nominal and ordinal data were expressed as percentages. Interval data were expressed as mean ± standard deviation (normal distribution) or median (lower–upper quartiles). The distribution of variables was evaluated by the Shapiro–Wilk test and the quantile–quantile (Q-Q) plot. In order to compare two groups with SHBG ≥ 26.1 nmol/L and SHBG <26.1 nmol/L, the Student *t*-test for independent data or the Mann–Whitney *U*-test was used according to the data distribution. The homogeneity of variances was assessed by the Fisher–Snedecor test. The correlation between SHGB levels and other variables was assessed using the Spearman rank correlation coefficient ρ. The correction for confounding variables was done with the Spearman rank partial correlation coefficient (package ‘*ppcor*' in R).

## Results

There were 52 (59.8%) PCOS women with SHBG levels below the lower limit of the reference range and 35 women (40.2%) over the limit (Table 1).

**Table 1:** Characteristics of the study subgroups.

	**SHBG LOW (*N =* 52)**	**SHBG HIGH (*N =* 35)**	***p*-value**
Age (years)	26 ± 5	25 ± 6	0.58
Body weight (kg)	83.0 (68.1–100.8)	63.1 (56.7–85.9)	< 0.01
BMI (kg/m^2^)	31.9 (24.6–36.7)	22.4 (20.6–30.8)	< 0.01
Overweight, N (%)	6 (11.5)	3 (8.6)	< 0.01
Obesity, N (%)	32 (61.5)	10 (28.6)	
Fat mass (kg)	34.1 (22.5–48.6)	18.8 (17.7–32.1)	< 0.01
Fat mass (%)	42.1 (32.7–48.3)	29.4 (26.2–41.8)	< 0.05
Waist circumference (cm)	95.0 ± 18.0	82.1 ± 17.1	< 0.01
Glucose (mmol/L)	5.0 (4.7–5.8)	5.0 (4.9–5.3)	0.65
Insulin (μIU/ml)	11.6 (8.9–16.8)	9.2 (6.5–11.3)	< 0.05
HOMA-IR	2.67 (1.99–4.08)	2.01 (1.31–2.62)	< 0.05
Estradiol (pg/ml)	39.4 (27.4–59.0)	47.4 (33.5–83.4)	0.09
Testosterone (ng/ml)	0.68 (0.49–0.78)	0.70 (0.53–1.00)	0.06
DHEA-S (μg/ml)	2.86 ± 1.08	2.90 ± 1.26	0.88
Androstendione (ng/ml)	2.34 ± 1.17	2.58 ± 1.29	0.37
SHBG (nmol/ml)	16.3 (10.5–20.7)	40.6 (33.6–53.4)	–
FAI	4.15 (2.90–5.79)	1.56 (1.14–2.22)	< 0.001
FAI > 5.0, N (%)	17 (32.7)	2 (5.7)	< 0.01
Omentin-1 (ng/ml)	206 (142–282)	221 (161–322)	0.17
Adiponectin (μg/ml)	5.35 (3.46–8.46)	7.63 (5.56–8.49)	< 0.05
Leptin (ng/ml)	43.5 (26.3–62.3)	16.6 (10.0–50.4)	< 0.01
Apelin-36 (ng/ml)	1.22 (0.90–1.71)	1.64 (1.15–3.15)	< 0.05
Apelin-12 (ng/ml)	0.33 (0.23–1.23)	1.50 (0.26–3.30)	< 0.05
RBP-4 (ng/ml)	0.33 (0.23–1.23)	1.50 (0.26–3.30)	0.36

Mean ± standard deviation or median (lower quartile - upper quartile).

As was expected, the subgroup with SHBG levels below the lower limit of the reference range (<26.1 nmol/L) was characterized by higher median BMI, fat mass percentage, waist circumference, insulin levels, HOMA-IR, and FAI value (and increased FAI percentage), as well as more frequent occurrence of overweight and obesity than the subgroup with higher SHBG levels.

The median adiponectin, apelin-12, and apelin-36 levels were significantly lower, and leptin levels were significantly higher in the subgroup with SHBG levels below 26.1 nmol/L. There were no differences in median omentin-1 and RBP-4 levels between the study subgroups (Table 1).

We observed positive correlations between SHBG and omentin-1, adiponectin, apelin-36, and apelin-12 levels, as well as negative correlations between SHBG and leptin levels. There was no association between SHBG and RBP-4 levels (Table 2).

**Table 2:** Raw and corrected by anthropometric parameters correlations between SHBG and listed adipokines in PCOS women (correlation coefficients calculated according to ρ Spearman).

	**Raw**	**Partial correlation corrected for**		
		**BMI**	**Waist circumference**	**Fat percentage**
Omentin-1	0.242^*^	0.356^#^	0.352^#^	0.333^**^
Adiponectin	0.219^*^	0.071	0.007	0.102
Leptin	−0.309^**^	−0.126	−0.057	−0.171
Apelin-36	0.246^*^	0.114	0.087	0.142
Apelin-12	0.213^*^	0.041	0.002	0.076
RBP-4	0.129	0.047	0.036	0.056

* *p* < 0.05;

** *p* < 0.01;

# *p* < 0.001.

We also examined the confounding effect of BMI, waist circumference, and body fat percentage on these associations in models adjusted for the factors listed above. Significant interaction terms were found for BMI, waist circumference, and body fat percentage concerning the association between SHBG and omentin-1; BMI, waist circumference, and body fat percentage interaction terms (*p* should be interaction <0.001, <0.001, and <0.01, respectively) were not statistically significant for associations between SHBG and any of the other adipokines (Table 2).

## Discussion

To the best of our knowledge, this is the first study that assessed the correlation between SHBG and adipokines levels in women with PCOS. Our study showed significantly lower median adiponectin, apelin-12, and apelin-36 and higher leptin levels in the subgroup with low SHBG levels. In addition, positive correlations between SHBG and omentin-1, adiponectin, apelin-36, and apelin-12 levels, as well as negative correlations between SHBG and leptin levels, were found. However, after adjustment by BMI, waist circumference, and body fat percentage, the only correlation between SHBG and omentin-1 levels remained significant. The obtained results are ambiguous and constitute a pilot study to further research the relationship between hormonal dysfunction of the adipose tissue and disturbances of hepatic SHBG synthesis in PCOS. At the level of experimental studies, only the influence of adiponectin on the signaling pathway of SHBG synthesis has been investigated. The study carried out on human liver cells showed that adiponectin affects hepatic enzymes activities, resulting in decreased hepatic lipid content due to the inhibition of lipogenesis and the stimulation of fatty acid oxidation and also increased hepatocyte nuclear factor 4 (HNF-4α) expression. All these mechanisms increase hepatic SHBG synthesis (5). It seems that leptin indirectly decreased HNF-4α expression by the stimulation of pro-inflammatory cytokines release (21). However, there is a lack of experimental studies confirming this hypothesis. In addition, leptin stimulates inflammation in the course of NAFLD, which may be an important mechanism inhibiting the synthesis of SHBG (12). As we have previously shown, the hyperandrogenism impairment of hormonal stroma adipose tissue function, secondary to insulin resistance, is independent of nutritional status. Meanwhile, changes in adiponectin release reflecting the hormonal dysfunction of adipocytes are dependent on the excess fat accumulation only (6–11, 13, 14). However, our study showed a lack of association between SHBG and adiponectin and also leptin levels after adjustment by BMI, waist circumference, and fat percentage. Similar results in relation to leptin were described in the study including non-diabetic elderly men, but the relationship between SHBG and adiponectin persisted after adjustment by BMI and waist circumference (22). The significant association between SHBG and adiponectin levels remained after adjustment for body composition assessed by dual-energy X-ray absorptiometry in young men, but the association with leptin was lost (23). Meanwhile, in a large sample of middle and old-aged Taiwanese men, the significant correlations between SHBG and both adiponectin and leptin levels remained after adjustment by BMI (11). The discrepancies may be explained both by sex and ethnic variations. However, data from the Study of Women's Health Across the Nation (SWAN) that included a large sample of white, African American, Chinese, Japanese, and Hispanic midlife women showed that the association between SHBG and leptin levels was substantially attenuated after adjustment for fat mass, but positive associations between SHBG and high molecular weight (HMW) adiponectin levels were quite robust after adjustment for fat mass and waist circumference (9). The differences between this study and our results may be the effect of measurement other than the HMW form of adiponectin total in our study. Another reason for the loss of correlation between SHBG and both leptin and adiponectin levels after adjustment by anthropometrics parameters in PCOS women may be the changes in adipokines profiles observed in PCOS. Although we did not observe any differences between RBP-4 and omentin-1 levels between subgroups with low and high SHBG levels, we observed a positive association between SHBG and omentin-1 levels, and this association remained significant after adjustment for BMI, waist circumference, and fat percentage. As we have previously shown, the hyperandrogenism impairment of hormonal stroma adipose tissue function, secondary to insulin resistance in PCOS, is independent of nutritional status, while changes in adiponectin release reflecting the hormonal dysfunction of adipocytes were only dependent on the excessive fat accumulation (24). Thus, the association between SHBG and omentin-1 levels may be the effect of developing a vicious circle of disease in PCOS, where adipose tissue hormonal dysfunction is the cause of hormonal disorders typical for PCOS, and these disorders may intensify the hormonal dysfunction of adipose tissue. On the other hand, the lack of association between SHBG and RBP-4 levels can be explained by our earlier observations that showed decreasing RBP-4 release along with increasing insulin resistance and hormonal disturbances in PCOS women with obesity (16). Our study has shown higher levels of apelin-12 and apelin-36 in the subgroup with high SHBG levels and positive correlations between SHBG and both apelin-12 and apelin-36 and their loss after adjustment for BMI, waist circumference, and fat percentage. It has been suggested that apelin synthesis is stimulated by insulin, while apelin inhibits insulin secretion. In addition, our previous study showed a positive association between both apelin-12 and apelin-36 and adiponectin levels and a negative association with leptin levels in PCOS (17). This once again supports the hypothesis that the effect of hormonal disturbances in the adipose tissue on SHBG synthesis in PCOS is quite complex. Further experimental and clinical studies are necessary to establish the effect of adipokines on SHBG synthesis and release.

The limitation of our study is the size of the study subgroups and the lack of a control group without PCOS. Moreover, the distribution of body fat and its visceral deposits were not directly assessed using DEXA or CT. Furthermore, we did not assess fatty liver and its effect on the association between SHBG and adipokines. In addition, in our study, only the interrelations between SHBG and selected adipokines were analyzed.

## Conclusion

Our results show associations between circulating SHBG and adipokine levels in women with PCOS and support the role of hormonal dysfunction of the adipose tissue in the pathogenesis of PCOS.

## Data availability statement

The raw data supporting the conclusions of this article will be made available by the authors, without undue reservation.

## Ethics statement

The studies involving human participants were reviewed and approved by the Bioethical Committee Medical University of Silesia. The patients/participants provided their written informed consent to participate in this study.

## Author contributions

Conceptualization: DC and MO-G. Methodology: DC, GF, and MO-G. Formal analysis and visualization: AO. Investigation: GF and PM. Resources and writing—original draft preparation: DC, PK, and LM. Data curation and funding acquisition: MO-G. Writing—review and editing: DC, AO, GF, PK, LM, PM, JC, and MO-G. Supervision: JC and MO-G. Project administration: PM. All authors contributed to the article and approved the submitted version.
